# Data source profile reporting by studies that use routinely collected health data to explore the effects of drug treatment

**DOI:** 10.1186/s12874-023-01922-8

**Published:** 2023-04-20

**Authors:** Wen Wang, Mei Liu, Qiao He, Mingqi Wang, Jiayue Xu, Ling Li, Guowei Li, Lin He, Kang Zou, Xin Sun

**Affiliations:** 1 Chinese Evidence-based Medicine Center and Cochrane China Center, West China Hospital, Sichuan University, 37 Guo Xue Xiang, Chengdu, 610041 Sichuan China; 2 NMPA Key Laboratory for Real World Data Research and Evaluation in Hainan, Chengdu, China; 3 Sichuan Center of Technology Innovation for Real World Data, Chengdu, China; 4 Department of Health Research Methods, Evidence and Impact, McMaster University, Hamilton, ON L8S 4L8 Canada; 5 Center for Clinical Epidemiology and Methodology, Guangdong Second Provincial General Hospital, Guangzhou, 510317 Guangdong China; 6 Biostatistics Unit, Research Institute at St. Joseph’s Healthcare Hamilton, Hamilton, ON L8N 4A6 Canada; 7 Intelligence Library Center, West China Hospital, Sichuan University, Chengdu, 610041 Sichuan China

**Keywords:** Routinely collected health data, Drug treatment effect, Reporting characteristics

## Abstract

**Background:**

Routinely collected health data (RCD) are important resource for exploring drug treatment effects. Adequate reporting of data source profiles may increase the credibility of evidence generated from these data. This study conducted a systematic literature review to evaluate the reporting characteristics of databases used by RCD studies to explore the effects of drug treatment.

**Methods:**

Observational studies published in 2018 that used RCD to explore the effects of drug treatment were identified by searching PubMed. We categorized eligible reports into two groups by journal impact factor (IF), including the top 5 general medical journals (NEJM, Lancet, JAMA, BMJ and JAMA Internal Medicine) and the other journals. The reporting characteristics of the databases used were described and compared between the two groups and between studies citing and not citing database references.

**Results:**

A total of 222 studies were included, of which 53 (23.9%) reported that they applied data linkage, 202 (91.0%) reported the type of database, and 211 (95.0%) reported the coverage of the data source. Only 81 (36.5%) studies reported the timeframe of the database. Studies in high-impact journals were more likely to report that they applied data linkage (65.1% vs. 20.2%) and used electronic medical records (EMR) (73.7% vs. 30.0%) and national data sources (77.8% vs. 51.3%) than those published in other medical journals. There were 137/222 (61.7%) cited database references. Studies with database-specific citations had better reporting of the data sources and were more likely to publish in high-impact journals than those without (mean IF, 6.08 vs. 4.09).

**Conclusions:**

Some deficits were found in the reporting quality of databases in studies that used RCD to explore the effects of drug treatment. Studies citing database-specific references may provide detailed information regarding data source characteristics. The adoption of reporting guidelines and education on their use is urgently needed to promote transparency by research groups.

**Supplementary Information:**

The online version contains supplementary material available at 10.1186/s12874-023-01922-8.

## Background

Routinely collected health data (RCD), including electronic medical records, health administrative data, and registries, are important resource for observational studies exploring the treatment effects of medicines [1–4]. These data contain information on drug exposures and outcomes that are essential for pharmacoepidemiology studies [1, 5]. The added support of information technologies that enable the storage of large datasets, including details on drug use, clinical management, laboratory test results, and patient outcomes, has led to a proliferation in observational pharmacoepidemiology studies in recent decades [3, 6, 7].

The increasing use of RCD has motivated the REporting of studies Conducted using Observational Routinely collected Data (RECORD) statement and its extension specific to pharmacoepidemiology (RECORD-PE) [1]. However, there are growing concerns about observational studies that use RCD to assess drug treatment effects [8–11], including the quality of the data used [12–15]. Complete and adequate reporting of data source profiles allows readers to effectively evaluate data quality and understand a study’s strengths and limitations. Meanwhile, poor reporting limits the assessment of scientific validity and leads to a misguided translation of research findings. Recently, concerns regarding the reporting of data source profiles have arisen.

The reporting of data source profiles among RCD studies exploring drug treatment remains unclear. Previous studies have shown that underreporting of data sources is common [16–18]. However, many of these studies were either outdated or used a small sample size [17–19], and none focused on research exploring the effects of drug treatment. For example, a literature review of 25 studies that used RCD for pharmacovigilance found that only 44% reported the type of data source [17]. Another review of 124 RCD studies published in 2012 showed that 28.2% did not report the type of database [18].

Detailed data source profiles are often limited in studies exploring the effects of drug treatment due to space restrictions. A published study or website including a data source profile can provide important information on data resources, data linkage, coverage, and the timeframe of the database [20, 21]. Citing a reference with a database profile helps to improve research transparency and strengthen scientific validity. The RECORD and RECORD-PE statements recommend referencing studies on data linkage and database validation [1, 5]. However, the method by which investigators cite database-specific references and whether citing references helps to improve reporting and publication remains uncertain. To date, no study has systematically examined the issue of database reporting, so a thorough investigation is strongly needed.

The current study was part of a major research project investigating the quality of the reporting and methods of observational studies using RCD to explore the effects of drug treatment. This project was conceptualized in early 2019 and has previously published the results on reporting of abstracts [16]. By providing empirical evidence about the present state of quality reporting, this study aims to inform recommendations on data source reporting and improve the transparency of observational studies of the effects of drug treatment using RCD.

## Methods

### Eligibility of studies

Observational studies that exclusively used RCD to explore the effects of drug treatment, including effectiveness, safety, or both, were included in the analysis. RCD was defined as data that were generated for administrative or clinical purposes without a priori research goals [1, 5]. Typical RCD includes electronic medical records (EMR), administrative claims data, safety surveillance databases, and pharmacy data [1, 7]. Studies that could not confirm whether the data resources were collected independently of prior research goals or that used at least one actively collected data element for research purposes were excluded.

### Literature search and selection process

Studies published between January 1 and December 31, 2018, were identified in PubMed using RCD-specific search terms. The National Library of Medicine’s search criteria regarding electronic health records were also used [22]. The detailed search strategy was described previously [16].

The sample size was calculated based on the number of factors that could be associated with study quality. Seven characteristics with eleven categories were considered as independent variables [23–25], resulting in a sample of 220 [16]. The journals were stratified into those published in the top five general medical journals and those published in lower-level medical journals according to the impact factor from the Institute for Scientific Information (ISI) Web of Knowledge Journal Citation Reports in 2018. The top five general medical journals, *BMJ, Journal of the American Medical Association, JAMA Internal Medicine, Lancet*, and *New England Journal of Medicine*, were those with the highest number of citations in 2018. All studies published in the top five general medical journals and a random sample of studies published in the lower-level medical journals were included. Detailed information on the sample size estimation and selection process was summarized previously [16].

### Data extraction

The general study characteristics and the reporting of database characteristics by each eligible study were collected from the full text, including the title and abstract. Information regarding the database characteristics in the titles and abstracts was not documented. Three study investigators (XS, WW, and ML) conducted a review of existing literature and guidance documents for routinely collected health data (e.g., RECORD, RECORD-PE, and others released by ISPOR, AHRQ) and developed the initial data extraction forms [1, 5, 26, 27]. The whole research team then brainstormed for additional items and developed a multidisciplinary research team that included one pharmacoepidemiology expert, two people who routinely conduct health data research, and two clinical epidemiologists to determine the importance of each item and reach a consensus about what items to include or exclude.

For general study characteristics, information on the specific disease, source of funding, number of participants, involvement of a methodologist, and type of outcomes were collected. For database characteristics, information related to database linkage, type of databases (EMR, claims data, or RCD not specified), name of the database, geographic region of the database, data source (i.e., inpatient records, outpatient records), variables collected (i.e., demographics, diagnosis, laboratory and microbiology tests, prescription, surgery), coverage and time span of the data source was collected. Information relating to database characteristics was documented based on the database descriptions in the included studies. For example, a study was considered to report data linkage if related words, such as “linked” and “linkage”, were used in the text. Studies citing references relating to the database in the Methods section were also documented. To fully capture information on database characteristics, information from relevant citations was also abstracted and reviewed.

### Data analysis

Descriptive analysis was used to describe the reporting characteristics of data sources in the included studies. Reporting characteristics, including data linkage across databases, types of data source, database name, database coverage, geographic region, data resources, data collection information, the time span of the data source, and population coverage were collected. Categorical variables were summarized as numbers (percentages), and continuous variables were summarized as the mean (standard deviation) or median (interquartile range, IQR).

The reporting of database characteristics was compared between studies published in the top five general medical journals and those published in other medical journals. The reporting quality and journal impact factor (IF) (provided by the 2018 Journal Citation Report) were also compared between studies that cited and did not cite database references. We used chi-squared test or Fisher’s exact test to compare categorical variables and the t test or nonparametric Wilcoxon’s test to compare continuous variables. The two tailed significance level was set at *P* < 0.05. Data analyses were performed using Stata/SE (version 14.0).

## Results

### Study characteristics

A total of 222 studies were included in the analysis (Supplementary Table 1). A flowchart of study identification, screening and inclusion is shown in Supplementary Fig. 1. The median number of participants in all included studies, the top five general medical journals, and other medical journals were 17,961 [interquartile range (IQR), 2,495–92,366], 154,162 [IQR, 58,994–289,469], and 15,597 [IQR, 1,925–80,198], respectively. Of the included studies, 114 (51.3%) received funding from a nonprofit organization, and 41 (18.4%) received industry funding. Detailed information on the study characteristics was described previously [16].

### Reporting characteristics of the data sources

Of the 222 included studies, 53 (23.9%) reported the use of data linkage, and of these, 22 (41.5%) reported the linkage methods used (Table 1). Most (202/222; 91.0%) reported the type of databases used, and the majority (211/222; 95.0%) reported coverage of the data source. The database name was reported by 195 (87.8%) studies, of which 89 (40.1%) specified the type of data source in the name. Of the included studies, 130 (58.6%) reported information on the data resource, 151 (68.0%) reported information on the data collected, and 81 (36.5%) included the timeframe of the database. Studies published in the top five medical journals were more likely than those published in lower-level journals to indicate the use of data linkage (63.1% and 20.2%, respectively; p < 0.001) (Table 1).

**Table 1:** Reporting characteristics of data sources of the included studies

Reporting item	Total	Journal type		
	(n = 222)	Top 5 general medicine (n = 19)	Non-top-5 general medicine (n = 203)	*P* value
**Linkage between data sources**, ***n*(%)**	53(23.9)	12(63.1)	41(20.2)	< 0.001
Reporting the methods of linkage, *n* (%)	22(41.5)	4(33.3)	18(43.9)	0.740
**Type of data source reported**, ***n*(%)**	202(91.0)	18(94.7)	184(90.6)	> 0.999
**Name of database reported**, ***n*(%)**	195(87.8)	19(100.0)	176(86.7)	0.139
Full name	66(29.7)	6(31.6)	60(29.6)	> 0.999
Abbreviation name	4(1.8)	0(0.0)	4(2.0)	
Both full and abbreviation name	125(56.3)	13(68.4)	111(54.7)	
***Whether the name of database include the type of data source, n (%)***				0.282
Yes	89(40.1)	6(31.6)	83(47.2)	
Partly*	20(9.0)	4(21.1)	16(9.1)	
Unclear^†^	25(11.3)	3(15.8)	22(12.5)	
Not include	61(27.5)	6(31.6)	55(31.3)	
**Coverage of data source**, ***n*(%)**	211(95.0)	18(94.7)	193(95.1)	> 0.999
**Categories of specific country**, ***n*(%)**	206(92.8)	19(100.0)	187(92.1)	0.372
**Data resource**‡, ***n*(%)**	130(58.6)	15(78.9)	115(56.7)	0.100
**Data collected**^**§**^, ***n*(%)**	151(68.0)	17(89.5)	134(66.0)	0.066
**Timeframe of database**, ***n*(%)**	81(36.5)	6(31.6)	75(36.9)	0.829
*Timeframe of database, median (years)*	22(12, 23)	30(30, 30)	22(12, 23)	0.005
**Population coverage**, ***n*(%)**	137(61.7)	13(68.4)	124(61.1)	0.702

* Study involved multiple databases, but only a part of database contained information related to the type of data source

^†^ The name of the data source containing wording which did not clarify the type of data source, such as “clinical practice”, “health”, and “health information”

‡ Data resource included inpatients records, outpatients records, prescription records, etc.

^**§**^ Data collected such as demographics, diagnosis, laboratory and microbiology tests, prescription, operation, etc.

**Fig. 1:**
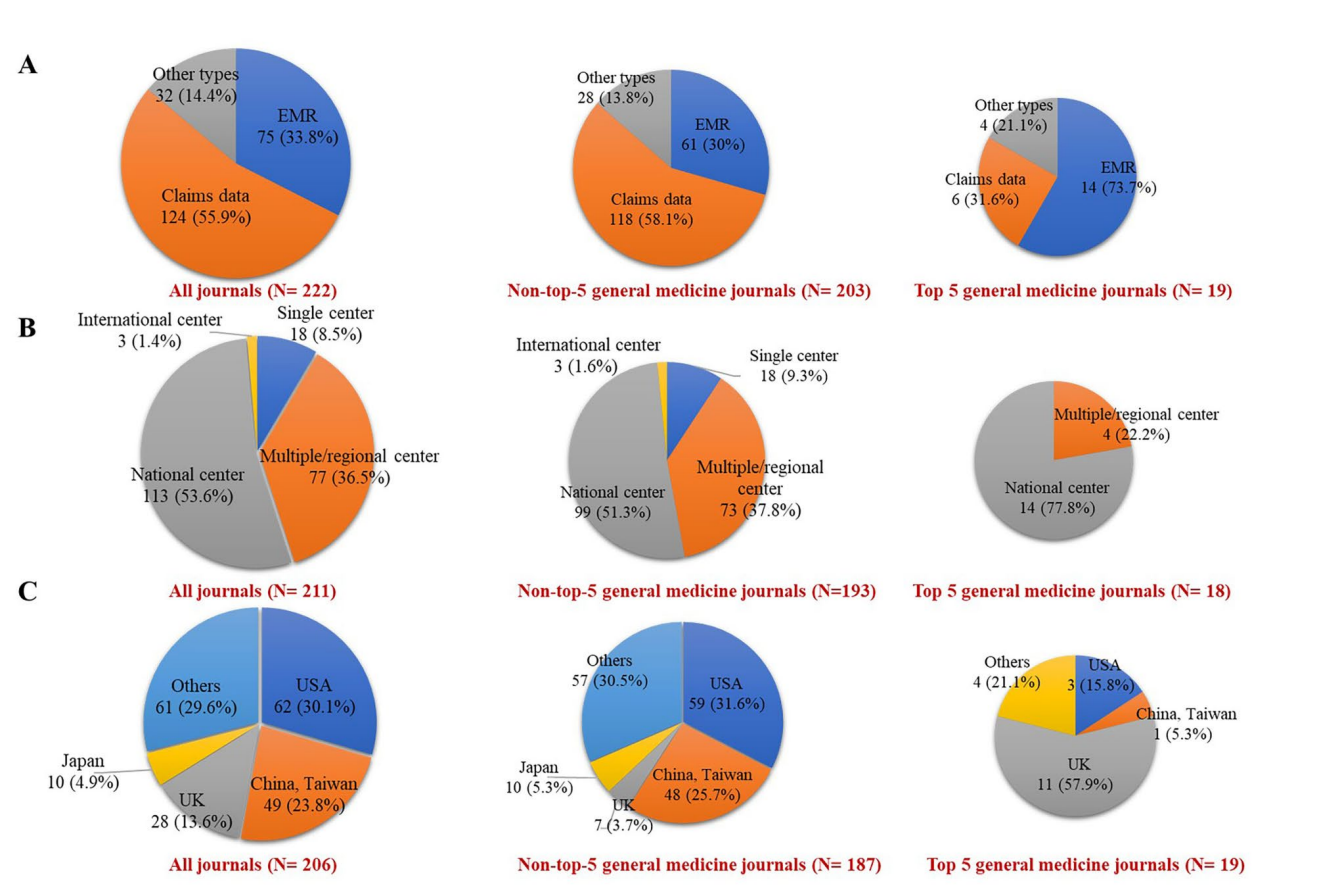
Characteristics of data sources of the included studies. (**A**) Type of database; (**B**) Coverage of data source; (**C**) Country of origin EMR: electronic medical record

Of all included studies, the most common database type was claims data (55.9%). The proportions of studies that used national data sources, multiple center or regional data sources, and single center data sources were 55.9%, 33.8%, and 14.4%, respectively. The largest percentage (30.1%) of studies used data from the United States, followed by China and Taiwan (23.8%) and the United Kingdom (UK) (13.6%) (Fig. 1A–C). A larger proportion of studies published in the top five general medical journals than those published in lower-level medical journals used EMR (73.7% and 30.0%, respectively; *p* < 0.001). Of 19 studies published in the top five general medical journals, 57.9% used data from the UK (Fig. 1A–C). The most common databases used among studies in the top five general medical journals and lower-level journals were the United Kingdom Clinical Practice Research Datalink (CPRD) (47.3%) and the Taiwan National Health Insurance Research Database (NHIRD) (25.7%), respectively.

### Citing database references

Of the 222 studies, 137 (61.7%) cited database-specific references. A total of 71 (32.0%) studies exclusively cited studies published in journals, 50 (22.5%) exclusively cited other reference types, such as websites and statistical files, and 16 (7.2%) cited both types of references (Table 2). Of 87 studies citing studies published in journals, 33 (14.9%) referenced database studies with detailed descriptions about the data source profile, 24 (10.8%) referenced validation studies, and 46 (20.7%) referenced case studies (Table 2). Of 137 studies citing database-specific references, 15 (78.9%) were published in the top five general medicine journals, while 122 (60.1%) were published in other journals. A larger proportion of studies published in the top five general medical journals than those published in lower-level journals cited references published in journals (63.2% and 36.9%, respectively; *p* = 0.046) and references specific to the data source profile (36.8% and 12.8%, respectively; *p* = 0.012) (Table 2).

**Table 2:** Citing database references among included studies

Reporting item	Total	Journal type		
	(n = 222)	Top 5 general medicine (n = 19)	Non-top-5 general medicine (n = 203)	*P* value
**Citing database references**, ***n*(%)**	137(61.7)	15(78.9)	122(60.1)	0.171
***Type of references, n (%)***				0.052
Exclusively cited studies published in journals	71(32.0)	8(42.1)	63(31.0)	
Exclusively cited other type of reference*	50(22.5)	3(15.8)	47(23.2)	
Both type of reference	16(7.2)	4(21.1)	12(0.1)	
***Referenced studies published in journals, n (%)***	87(39.2)	12(63.2)	75(36.9)	0.046
Study regarding data source profile	33(14.9)	7(36.8)	26(12.8)	0.012
Validation study	24(10.8)	5(26.3)	19(9.4)	0.039
Case study	46(20.7)	5(26.3)	41(20.2)	0.556
**Published time of referenced studies** ^†^	2015 (2012, 2016)	2015 (2010, 2015)	2015 (2012, 2017)	—

* Other types of reference such as website and statistical files

†We recorded the lasted time if multiple published times were reported

Reporting quality was generally better among studies that cited database-specific references (Table 3). For example, 105 (76.6%) studies that cited database-specific references reported data resource information, while only 25 (29.4%) of those that did not cite database-specific references reported data resource information. In addition, while 79.6% of studies citing database-specific references reported data collection information, only 49.4% of studies not citing database-specific references reported this information (Table 3).

**Table 3:** Reporting characteristics of studies citing or not citing database references

Reporting item	Studies not citing references	Studies citing database references		
	(n = 85)	Any type of reference (n = 137)	Published studies (n = 87)	Other type of reference (n = 66)
**Linkage between data sources, n (%)**	15(17.6)	38(27.7)	20(23.0)	20(30.3)
**Type of data source reported, n (%)**	79(92.9)	123(89.8)	80(92.0)	57(86.4)
**Name of database reported, n (%)**	70(82.4)	125(91.2)	80(92.0)	60(90.9)
**Coverage of data source, n (%)**	77(90.6)	134(97.8)	86(98.9)	64(97.0)
**Categories of specific country, n (%)**	77(90.6)	129(94.2)	83(95.4)	63(95.5)
**Data resource**^**§**^, **n (%)**	25(29.4)	105(76.6)	70(80.5)	51(77.3)
Data collected**, n (%)	42(49.4)	109(79.6)	76(87.4)	48(72.7)
**Timeframe of database, n (%)**	18(21.2)	63(46.0)	47(54.0)	26(39.4)

**Fig. 2:**
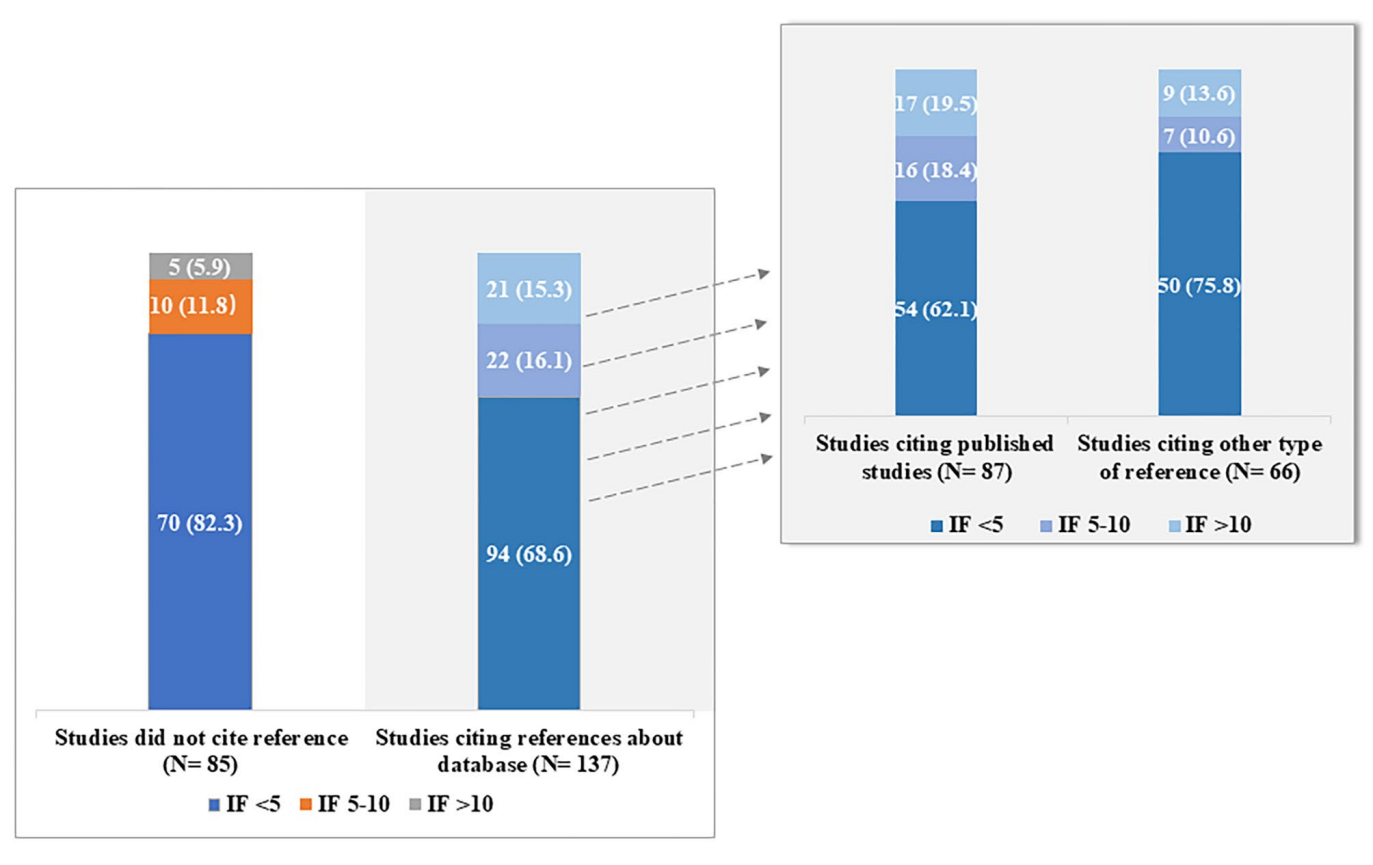
Journal impact factor among studies citing or not citing references regarding database. IF: impact factor

Studies that cited database-specific references were more likely than those that did not cite database-specific references to publish in high-impact journals (mean IF, 6.08 and 4.09, respectively; *p* = 0.006). The proportions of studies published in journals with an IF > 10 among studies with and without citing database-specific references were 15.3% and 5.9%, respectively (Fig. 2).

## Discussion

### Main findings and interpretations

RCD has been increasingly used for exploring drug treatment effects; however, growing concerns have arisen about the potential risk of bias induced by data quality. Since RCD are developed without a priori research purpose, assessing whether the data elements contained within the data source are sufficient to address the research questions is essential. Transparent and detailed reporting of data source profiles may facilitate users of research to assess the risk of bias and optimally interpret the research findings. However, our study found some deficiencies in the reporting of data sources, even by studies published in the top five general medical journals. For example, only 41.5% of studies that used data linkage approaches reported the linkage methods used, and almost two-thirds of studies did not include the database timeframe. Similar to our findings, a survey of 124 RCD studies published in 2012 found that only 29.3% of studies adequately reported data linkage [18]. Another study of 56 urological manuscripts published in 2014 showed that 48.2% reported the geographic region of the database, and none reported the methods used to link the data [19].

This study found that data source characteristics differed between those studies published in the top five general medical journals and those published in lower-level medical journals. Those published in the top medical journals were more likely to use EMR and national data sources, while those published in lower medical journals more often used claims data. Administrative claims data often lack important information, such as laboratory results and over-the-counter drug use [28]. The absence of these data can limit the extent to which studies on the effects of drug treatment can address a prognostic imbalance.

Our study also found that 61.7% of studies cited references regarding the database profile. Studies that cited database references often had a higher quality of data source reporting and were more likely to publish in high-impact journals than those without. The potential reason may be that citing a database reference can provide important information regarding the database profile, which helps to increase the credibility of evidence generated from these data [29–31]. No study, however, has addressed this issue.

### Implications

Adequate and transparent reporting is key to producing valid and reliable evidence to inform decision-making, and our study highlights potential areas for improvement. Researchers should include information regarding the database profile to improve readers’ understanding and assess the quality of the data sources. Since detailed descriptions of the data source profile may be restricted by word count limitations, citing a database reference can provide important information regarding the database profile and allow researchers and readers to critically evaluate potential bias relating to the data quality. Several organizations have developed searchable repositories of information on database profiles, including the Health Data Research UK and the DARWIN project from the European Medicines Agency [32–34]. RECORD and RECORD-PE statements are important to improve the reporting quality of RCD studies. The adoption of reporting guidelines and education on their use is urgently needed to promote the transparency of studies using RCD to explore the effects of drug treatment.

### Strengths and limitations

This study has several strengths. First, it included several representative studies using RCD to explore the effects of drug treatment. Previous studies had a small sample size and were restricted to specific topics and journals. Second, rigorous methods were used to thoroughly identify eligibilities, and standardized forms were developed to improve the accuracy of extraction.

Some caveats, however, should be considered in this study. First, it only included studies published in 2018. Thus, the findings may not be generalizable to other years. However, the practice of using RCD to assess the effects of drug treatment is unlikely to have changed significantly over a relatively short period. To further confirm this, the reporting quality of a sample of studies published in 2021 was investigated. PubMed was searched for RCD studies published in 2021, and the reports were placed in chronological order of their publication. The first 20 reports that met the eligibility criteria were selected, and the reporting quality was comparable to those published in 2018. All the studies reported the type of database, and 90.0% reported the data source. Only 25% of the studies reported that they used data linkage, of which 50% reported the methods used (supplementary Table 2). Studies that were labeled as a registry without specifying whether the data were collected for administrative or research purposes were excluded. The definition of a registry and the approach used to collect data for registries varies substantially [35, 36]. In this study, registries were defined as those in which RCD was collected for administrative purposes. Third, this study only included 19 articles published in the top five general medical journals, which may not be representative of all studies published in these journals. Fourth, the reporting of data sharing or exchanging was not investigated by this study.

## Conclusions

This study found some deficiencies in the reporting of data sources, such as linkage methods and timeframe. The reporting quality and characteristics of the data sources differed between studies published in the top five general medical journals and other journals. Studies citing database-specific references may provide detailed information regarding data source characteristics and are more likely to publish in high-impact journals. The adoption of reporting guidelines and education on their use is urgently needed to promote transparency.

## Electronic supplementary material

Below is the link to the electronic supplementary material.

Supplementary Material 1

Supplementary Material 2

Supplementary Material 3

## Data Availability

The datasets used and/or analysed during the current study available from the corresponding author on reasonable request.

## List of abbreviations

RCD: Routinely collected health data
RECORD: REporting of studies Conducted using Observational Routinely-collected health data statement
RECORD-PE: REporting of studies Conducted using Observational Routinely-collected health data statement for pharmacoepidemiology
EMR: Electronic Medical Record
IQR: Interquartile Range
IF: Impact Factor
NHIRD: National Health Insurance Research Database
CPRD: Clinical Practice Research Datalink
