# Supplementary material for: Data source profile reporting by studies that use routinely collected health data to explore the effects of drug treatment
Source: BMC Med Res Methodol. 2023 Apr 20;23:95. doi: 10.1186/s12874-023-01922-8 (PMC10120171; doi:10.1186/s12874-023-01922-8)
Supplement: Supplementary file 1 — Supplementary Material 1 [file 12874_2023_1922_MOESM1_ESM.docx]

**Supplementary table 1 List of included studies**

| **First Author** | **Title** | **Journal** | **Date** | **PMID** |
| --- | --- | --- | --- | --- |
| **Published in 2018** | |  |  |  |
| **Top 5 general medicine** **journals** | |  |  |  |
| Abrahami D | Dipeptidyl peptidase-4 inhibitors and incidence of inflammatory bowel disease among patients with type 2 diabetes: population based cohort study | BMJ | 21-Mar | 29563098 |
| Ray WA | Association of oral anticoagulants and proton pump inhibitor cotherapy with hospitalization for upper gastrointestinal tract bleeding | JAMA Intern Med | 4-Dec | 30512099 |
| Sheppard JP | Benefits and harms of antihypertensive treatment in low-risk patients with mild hypertension | JAMA Intern Med | 1-Dec | 30383082 |
| Douros A | Sulfonylureas as second line drugs in type 2 diabetes and the risk of cardiovascular and hypoglycaemic events: population based cohort study | BMJ | 18-Jul | 30021781 |
| Chang HY | Association between sodium-glucose cotransporter 2 inhibitors and lower extremity amputation among patients with type 2 diabetes | JAMA Intern Med | Sep | 30105373 |
| Abrahami D | Incretin based drugs and risk of cholangiocarcinoma among patients with type 2 diabetes: population based cohort study | BMJ | 5-Dec | 30518618 |
| Vinogradova Y | Risks and benefits of direct oral anticoagulants versus warfarin in a real world setting: cohort study in primary care | BMJ | 4-Jul | 29973392 |
| Lazarus B | Association of metformin use with risk of lactic acidosis across the range of kidney function: a community-based cohort study | JAMA Intern Med | 1-Jul | 29868840 |
| Gafoor R | Antidepressant utilisation and incidence of weight gain during 10 years' follow-up: population based cohort study | BMJ | 23-May | 29793997 |
| Richardson K | Anticholinergic drugs and risk of dementia: case-control study | BMJ | 25-Apr | 29695481 |
| Ramos R | Statins for primary prevention of cardiovascular events and mortality in old and very old adults with and without type 2 diabetes: retrospective cohort study | BMJ | 5-Sep | 30185425 |
| Gomes T | Contributions of prescribed and non-prescribed opioids to opioid related deaths: population based cohort study in Ontario, Canada | BMJ | 29-Aug | 30158106 |
| Hicks BM | Angiotensin converting enzyme inhibitors and risk of lung cancer: population based cohort study | BMJ | 24-Oct | 30355745 |
| Patorno E | Cardiovascular outcomes associated with canagliflozin versus other non-gliflozin antidiabetic drugs: population based cohort study | BMJ | 6-Feb | 29437648 |
| Bouck Z | Frequency and associations of prescription nonsteroidal anti-inflammatory drug use among patients with a musculoskeletal disorder and hypertension, heart failure, or chronic kidney disease | JAMA Intern Med | 1-Nov | 30304456 |
| Park Y | Use of haloperidol versus atypical antipsychotics and risk of in-hospital death in patients with acute myocardial infarction: cohort study | BMJ | 28-Mar | 29592958 |
| Brar S | Association of angiotensin-converting enzyme inhibitor or angiotensin receptor blocker use with outcomes after acute kidney injury | JAMA Intern Med | 1-Dec | 30422153 |
| Wang MT | Association of cardiovascular risk with inhaled long-acting bronchodilators in patients with chronic obstructive pulmonary disease: a nested case-control study | JAMA Intern Med | 1-Feb | 29297057 |
| Crellin E | Trimethoprim use for urinary tract infection and risk of adverse outcomes in older patients: cohort study | BMJ | 9-Feb | 29438980 |
| **Non-top 5 general medicine journals** | | |  |  |
| Lemon LS | Methadone versus buprenorphine for opioid use dependence and risk of neonatal abstinence syndrome | Epidemiology | Mar | 29112519 |
| Dayan V | CABG and preoperative use of beta-blockers in patients with stable angina are associated with better cardiovascular survival | Braz J Cardiovasc Surg | Jan-Feb | 29617501 |
| Huang BT | No modifying effect of nutritional status on statins therapy in relation to all-cause death in older patients with coronary artery disease | Aging Clin Exp Res | Sep | 29260400 |
| Kosiborod M | Rates of myocardial infarction and stroke in patients initiating treatment with SGLT2-inhibitors versus other glucose-lowering agents in real-world clinical practice: results from the CVD-REAL study | Diabetes Obes Metab | Aug | 29569378 |
| Faour M | Low-dose aspirin is safe and effective for venous thromboembolism prophylaxis following total knee arthroplasty | J Arthroplasty | Jul | 29656974 |
| Newcomer JW | Hospitalization outcomes in patients with schizophrenia after switching to lurasidone or quetiapine: a us claims database analysis | BMC Health Serv Res | 4-Apr | 29618351 |
| Coleman CI | Effectiveness and safety of outpatient rivaroxaban versus warfarin for treatment of venous thromboembolism in patients with a known primary hypercoagulable state | Thromb Res | Mar | 29407625 |
| Brzozowska M | Analysis of survival of patients treated with vemurafenib, ipilimumab and dabrafenib for advanced skin melanoma in daily clinical practice (real-world data): retrospective analysis of patients treated under drug/reimbursement programmes in Poland in 2013- | Melanoma Res | Feb | 29120964 |
| Dörks M | Combined use of drugs inhibiting the renin-angiotensin system: prescribing patterns and risk of acute kidney injury in German nursing home residents | Clin Interv Aging | 7-Jun | 29872281 |
| Foch C | In utero drug exposure and hearing impairment in 2-year-old children a case-control study using the EFEMERIS database | Int J Pediatr Otorhinolaryngol | Oct | 30173984 |
| Cea-Soriano L | Safety of non-insulin glucose-lowering drugs in pregnant women with pre-gestational diabetes: a cohort study | Diabetes Obes Metab | Jul | 29498473 |
| Chiu AS | Recurrent falls among elderly patients and the impact of anticoagulation therapy | World J Surg | Dec | 29959494 |
| Liu AR | A matched case-control study to assess the association between non-steroidal anti-inflammatory drug use and thrombotic microangiopathy | PLoS One | 25-Aug | 30142223 |
| Kim YG | Sodium-glucose co-transporter-2 inhibitors and the risk of ketoacidosis in patients with type 2 diabetes mellitus: a nationwide population-based cohort study | Diabetes Obes Metab | Aug | 29569427 |
| Yen FS | Effects of metformin use on total mortality in patients with type 2 diabetes and chronic obstructive pulmonary disease: a matched-subject design | PLoS One | 5-Oct | 30286138 |
| Lai CC | Comparative effects of angiotensin-converting enzyme inhibitors and angiotensin ii receptor blockers on the risk of pneumonia and severe exacerbations in patients with COPD | Int J Chron Obstruct Pulmon Dis | 23-Mar | 29563786 |
| Cheng SY | Benzodiazepines and risk of pneumonia in schizophrenia: a nationwide case-control study | Psychopharmacology (Berl) | Nov | 30232530 |
| Tyerman Z | Preoperative statin use not associated with improved outcomes after ascending aortic repair | Semin Thorac Cardiovasc Surg | Winter | 30102969 |
| Wu CS | Psychotropic use and risk of stroke among patients with bipolar disorders: 10-year nationwide population based study | J Affect Disord | 15-Jan | 28964996 |
| Lentine KL | Antidepressant medication use before and after kidney transplant: implications for outcomes - a retrospective study | Transpl Int | Jan | 28771882 |
| Harrington R | The relationship between oseltamivir and suicide in pediatric patients | Ann Fam Med | Mar | 29531106 |
| Morgan CL | Impact of treatment with pioglitazone on stroke outcomes: a real-world database analysis | Diabetes Obes Metab | Sep | 29732718 |
| Nam YH | Nonsteroidal anti-inflammatory drug choice and adverse outcomes in clopidogrel users: a retrospective cohort study | PLoS One | 15-Mar | 29538453 |
| Coleman CI | Effectiveness and safety of rivaroxaban versus warfarin in patients with provoked venous thromboembolism | J Thromb Thrombolysis | Oct | 29881958 |
| Charlton B | Length of hospitalization and mortality for bleeding during treatment with warfarin, dabigatran, or rivaroxaban | PLoS One | 29-Mar | 29590141 |
| Meiser A | Inhalation sedation in subjects with ARDS undergoing continuous lateral rotational therapy | Respir Care | Apr | 29233852 |
| Shaheen AA | The impact of depression and antidepressant usage on primary biliary cholangitis clinical outcomes | PLoS One | 5-Apr | 29617396 |
| Knudsen-Baas KM | Antiepileptic and psychiatric medication in a nationwide cohort of patients with glioma who grade ii-iv | J Neurooncol | Dec | 30471051 |
| Choi JY | Incidence and risk factors for congestive heart failure in patients with early breast cancer who received anthracycline and/or trastuzumab: a big data analysis of the Korean health insurance review and assessment service database | Breast Cancer Res Treat | Aug | 29737474 |
| Kelley BP | Postoperative ketorolac in breast and body contouring procedures: a nationwide claims analysis | Plast Reconstr Surg | Oct | 30252810 |
| Huang CY | Effect of statin therapy on the prevention of new-onset acute coronary syndrome in patients with rheumatoid arthritis | Int J Cardiol | 15-Feb | 29174015 |
| Kallenbach L | Predictors and clinical outcomes of treatment intensification in patients with type 2 diabetes uncontrolled on basal insulin in a real-world setting | Endocr Pract | Sep | 29975575 |
| Tsai CH | Inhaled corticosteroids and the risks of low-energy fractures in patients with chronic airway diseases: a propensity score matched study | Clin Respir J | May | 29148205 |
| Chen CA | Survival benefit of patients with early-stage ovarian carcinoma treated with paclitaxel chemotherapeutic regimens | J Gynecol Oncol | Jan | 29185274 |
| Krafcik BM | Preoperative antiplatelet and statin use does not affect outcomes after carotid endarterectomy | Ann Vasc Surg | Jan | 29100876 |
| Chiu HY | Risk of psoriasis following terbinafine or itraconazole treatment for onychomycosis: a population-based case-control comparative study | Drug Saf | Mar | 29110252 |
| Tong L | Impact of delaying treatment intensification with a glucagon-like peptide-1 receptor agonist in patients with type 2 diabetes uncontrolled on basal insulin: a longitudinal study of a us administrative claims database | Diabetes Obes Metab | Apr | 29119712 |
| Tanaka S | Real-world evidence of raloxifene versus alendronate in preventing non-vertebral fractures in Japanese women with osteoporosis: retrospective analysis of a hospital claims database | J Bone Miner Metab | Jan | 28028633 |
| Ajayi T | Role of nucleoside/nucleotide analogues and low-dose hepatitis b immune globulin in prophylaxis of hepatitis b recurrence among cadaveric liver transplant recipients | Turk J Gastroenterol | Jan | 29391309 |
| Cho YY | Protective effect of metformin against thyroid cancer development: a population-based study in Korea | Thyroid | Jul | 29808777 |
| Coleman CI | Postthrombotic syndrome in patients treated with rivaroxaban or warfarin for venous thromboembolism | Clin Appl Thromb Hemost | May | 29514466 |
| Jerzak KJ | Prevention of carboplatin-induced hypersensitivity reactions in women with ovarian cancer | J Oncol Pharm Pract | Mar | 27856924 |
| Wu C | Assessing the risk for peripheral neuropathy in patients treated with dronedarone compared with that in other antiarrhythmics | Clin Ther | Mar | 29500139 |
| Loughlin AM | Effectiveness and tolerability of therapy with exenatide once weekly vs basal insulin among injectable-drug-naive elderly or renal impaired patients with type 2 diabetes in the United States | Diabetes Obes Metab | 14-Sep | 30220442 |
| Lin TK | Long-term effect of statins on the risk of new-onset osteoporosis: a nationwide population-based cohort study | PLoS One | 4-May | 29723231 |
| Hwang KT | Tamoxifen therapy improves overall survival in luminal a subtype of ductal carcinoma in situ: a study based on nationwide Korean breast cancer registry database | Breast Cancer Res Treat | Jun | 29383628 |
| Kawasaki R | Lipid-lowering medication is associated with decreased risk of diabetic retinopathy and the need for treatment in patients with type 2 diabetes: a real-world observational analysis of a health claims database | Diabetes Obes Metab | Oct | 29790265 |
| Biffi A | Antidepressants and the risk of arrhythmia in elderly affected by a previous cardiovascular disease: a real-life investigation from Italy | Eur J Clin Pharmacol | Jan | 29046942 |
| Lin HF | Correlation of the tamoxifen use with the increased risk of deep vein thrombosis and pulmonary embolism in elderly women with breast cancer: a case-control study | Medicine (Baltimore) | Dec | 30572423 |
| Pontes C | Analgesic use and risk for acute coronary events in patients with osteoarthritis: a population-based, nested case-control study | Clin Ther | Feb | 29398161 |
| Coleman CI | Effectiveness and safety of rivaroxaban vs warfarin in people with non-valvular atrial fibrillation and diabetes: an administrative claims database analysis | Diabet Med | Aug | 29663521 |
| Lu CH | Combination cox-2 inhibitor and metformin attenuate rate of joint replacement in osteoarthritis with diabetes: a nationwide, retrospective, matched-cohort study in Taiwan | PLoS One | 1-Feb | 29385156 |
| Tzeng NS | Magnesium oxide use and reduced risk of dementia: a retrospective, nationwide cohort study in Taiwan | Curr Med Res Opin | Jan | 28952385 |
| Beliaev AM | Low-dose erythropoietin treatment is not associated with clinical benefits in severely anaemic Jehovah's Witnesses: a plea for a change | Blood Transfus | Jan | 27893353 |
| Kim H | Change in ALT levels after administration of HMG-CoA reductase inhibitors to subjects with pretreatment levels three times the upper normal limit in clinical practice | Cardiovasc Ther | Jun | 29464863 |
| Lin XH | Risk factors for upper gastrointestinal bleeding in patients taking selective cox-2 inhibitors: a nationwide population-based cohort study | Pain Med | 1-Feb | 28460044 |
| Wu FJ | Increased risk of a herpes zoster attack in patients receiving androgen deprivation therapy for prostate cancer | Andrologia | Mar | 28786220 |
| Tolia VN | Morphine vs methadone treatment for infants with neonatal abstinence syndrome | J Pediatr | Dec | 30220442 |
| Gulati S | Risk of intracranial hemorrhage (rich) in users of oral antithrombotic drugs: nationwide pharmacoepidemiological study | PLoS One | 24-Aug | 30138389 |
| Yuan Z | Risk of lower extremity amputations in people with type 2 diabetes mellitus treated with sodium-glucose co-transporter-2 inhibitors in the USA: a retrospective cohort study | Diabetes Obes Metab | Mar | 28898514 |
| Lai SW | Synergistic effect of oral corticosteroids use on risk of hepatocellular carcinoma in high risk populations | Eur J Intern Med | Jun | 29555406 |
| Liang CY | Cardiovascular risk of sitagliptin in ischemic stroke patients with type 2 diabetes and chronic kidney disease: a nationwide cohort study | Medicine (Baltimore) | Dec | 30593182 |
| Frayne J | The relationship between pregnancy exposure to antidepressant and atypical antipsychotic medications and placental weight and birth weight ratio: a retrospective cohort study | J Clin Psychopharmacol | Dec | 30346334 |
| Douros A | Glucagon-like peptide 1 receptor agonists and the risk of incident diabetic retinopathy | Diabetes Care | Nov | 30150234 |
| Shaaban D | The effect of tumor necrosis factor inhibitor therapy on the incidence of myocardial infarction in patients with psoriasis: a retrospective study | J Dermatolog Treat | Feb | 27881030 |
| Kato H | Intravenous administration of tacrolimus stabilizes control of blood concentration regardless of cyp3a5 polymorphism in living donor liver transplantation: comparison of intravenous infusion and oral administration in early postoperative period | Transplant Proc | Nov | 30401377 |
| Bell KL | Preoperative opioids increase the risk of periprosthetic joint infection after total joint arthroplasty | J Arthroplasty | Oct | 30054211 |
| Tadokoro F | Association between sugammadex and anaphylaxis in pediatric patients: a nested case-control study using a national inpatient database | Paediatr Anaesth | Jul | 29947043 |
| Tamimi I | Acetylcholinesterase inhibitors and the risk of osteoporotic fractures: nested case-control study | Osteoporos Int | Apr | 29264626 |
| Ajam T | Effect of carvedilol vs metoprolol succinate on mortality in heart failure with reduced ejection fraction | Am Heart J | May | 29754646 |
| Tseng CH | Metformin is associated with decreased skin cancer risk in Taiwanese patients with type 2 diabetes | J Am Acad Dermatol | Apr | 29246826 |
| Arima R | Antidiabetic medication, statins and the risk and prognosis of non-endometrioid endometrial cancer in women with type 2 diabetes | Anticancer Res | Jul | 29970546 |
| Horstman H | Safety of long-term anticoagulation in patients with brain metastases | Med Oncol | 1-Mar | 29497873 |
| Li X | Apixaban 5 and 2.5 mg twice-daily versus warfarin for stroke prevention in nonvalvular atrial fibrillation patients: comparative effectiveness and safety evaluated using a propensity-score-matched approach | PLoS One | 27-Jan | 29373602 |
| Albrecht JS | Safety of antidepressant classes used following traumatic brain injury among Medicare beneficiaries: a retrospective cohort study | Drugs Aging | Aug | 30047070 |
| Hwang IC | Association between proton pump inhibitor use and the risk of pancreatic cancer: a Korean nationwide cohort study | PLoS One | 13-Sep | 30208110 |
| Andrew NE | Prescription of antihypertensive medication at discharge influences survival following stroke | Neurology | 27-Feb | 29386279 |
| Overbeek JA | Clinical effectiveness of liraglutide vs basal insulin in a real-world setting: evidence of improved glycaemic and weight control in obese people with type 2 diabetes | Diabetes Obes Metab | Sep | 29726082 |
| Li YR | Linagliptin and cardiovascular outcomes in type 2 diabetes after acute coronary syndrome or acute ischemic stroke | Cardiovasc Diabetol | 4-Jan | 29301579 |
| Ho CM | Comparative effectiveness of angiotensin-converting enzyme inhibitors and angiotensin ii receptor blockers in chemoprevention of hepatocellular carcinoma: a nationwide high-risk cohort study | BMC Cancer | 10-Apr | 29631561 |
| Rautiainen P | Glycemic control in adult type 1 diabetes patients with insulin glargine, insulin detemir, or continuous subcutaneous insulin infusion in daily practice | Diabetes Technol Ther | May | 29741925 |
| Liao WI | Valproic acid attenuates the risk of acute respiratory failure in patients with subarachnoid hemorrhage | QJM | 1-Feb | 29048544 |
| Dreyfus J | Clostridial collagenase ointment and medicinal honey utilization for pressure ulcers in us hospitals | J Med Econ | Apr | 29295637 |
| Van DalemJ | Risk of a first-ever acute myocardial infarction and all-cause mortality with sulphonylurea treatment: a population-based cohort study | Diabetes Obes Metab | Apr | 29171906 |
| Xue YH | Etoricoxib and diclofenac might reduce the risk of dementia in patients with osteoarthritis: a nation-wide, population-based retrospective cohort study | Dement Geriatr Cogn Disord | 29-Jun | 29953974 |
| Tang KT | Psoriatic patients with chronic viral hepatitis do not have an increased risk of liver cirrhosis despite long-term methotrexate use: real-world data from a nationwide cohort study in Taiwan | J Am Acad Dermatol | Oct | 29753054 |
| Shvartzman Y | Adjunctive antidepressants in bipolar depression: a cohort study of six- and twelve-months rehospitalization rates | Eur Neuropsychopharmacol | Mar | 29449055 |
| Kim MC | Statins increase the risk of herpes zoster: a propensity score-matched analysis | PLoS One | 15-Jun | 29902266 |
| Bedard NA | Preoperative opioid use and its association with early revision of total knee arthroplasty | J Arthroplasty | Jan | 28655175 |
| Lu CH | Lower risk of dementia with pioglitazone, compared with other second-line treatments, in metformin-based dual therapy: a population-based longitudinal study | Diabetologia | Mar | 29138876 |
| Peckham AM | All-cause and drug-related medical events associated with overuse of gabapentin and/or opioid medications: a retrospective cohort analysis of a commercially insured us population | Drug Saf | Feb | 28956286 |
| Zielen S | Sublingual immunotherapy provides long-term relief in allergic rhinitis and reduces the risk of asthma: a retrospective, real-world database analysis | Allergy | Jan | 28561266 |
| Generali E | Comparison of the risks of hospitalisation for cardiovascular events in patients with rheumatoid arthritis treated with tocilizumab and etanercept | Clin Exp Rheumatol | Mar-Apr | 29303702 |
| Schink T | Risk of ischemic stroke and the use of individual non-steroidal anti-inflammatory drugs: a multi-country European database study within the SIS project | PLoS One | Jan | 28548546 |
| Sobh AH | The role of aspirin and unfractionated heparin combination therapy immediately after total hip and knee arthroplasty | Orthopedics | 1-May | 29570760 |
| Lee WJ | Risk of serious bacterial infection associated with tumour necrosis factor-alpha inhibitors in children with juvenile idiopathic arthritis | Rheumatology (Oxford) | Feb | 28431162 |
| Shorbatli LA | Effectiveness of antibiotic therapy in pediatric patients with cat scratch disease | Int J Clin Pharm | Dec | 30446895 |
| Lin HF | Tamoxifen usage correlates with increased risk of Parkinson’s disease in older women with breast cancer: a case-control study in Taiwan | Eur J Clin Pharmacol | May | 29481993 |
| Mueller C | Associations of acetylcholinesterase inhibitor treatment with reduced mortality in Alzheimer’s disease: a retrospective survival analysis | Age Ageing | Aug | 29504234 |
| Garry EM | Comparative safety of pioglitazone versus clinically meaningful treatment alternatives concerning the risk of bladder cancer in older us adults with type 2 diabetes | Diabetes Obes Metab | Jan | 28661561 |
| Tam HW | Methotrexate might reduce ischemic stroke in patients with rheumatoid arthritis: a population-based retrospective cohort study | Int J Rheum Dis | Aug | 29372595 |
| Chiu HT | Effect of statin use on the risk of medically attended acute respiratory illness among influenza vaccinated elderly | Vaccine | 1-Oct | 30174239 |
| Aharaz A | Risk of lactic acidosis in type 2 diabetes patients using metformin: a case control study | PLoS One | 9-May | 29738540 |
| Lim DH | The risk of herpes zoster in patients with ankylosing spondylitis: analysis of the Korean national health insurance service - sample cohort database | Mod Rheumatol | Jan | 28548546 |
| Hochreiter M | Prolonged antibiotic prophylaxis after thoracoabdominal esophagectomy does not reduce the risk of pneumonia in the first 30 days: a retrospective before-and-after analysis | Infection | Oct | 29869780 |
| Lee HF | End-stage renal disease patients using angiotensin-converting enzyme inhibitors and angiotensin receptor blockers may reduce the risk of mortality: a Taiwanese nationwide cohort study | Intern Med J | Sep | 29808610 |
| Madden JM | De novo vitamin d supplement use post-diagnosis is associated with breast cancer survival | Breast Cancer Res Treat | Nov | 30039288 |
| Lin CC | Long-term use of valproic acid and the prevalence of cancers in bipolar disorder patients in a Taiwanese population: an association analysis using the national health insurance research database (NHIRD) | J Affect Disord | May | 29481993 |
| Korhonen MJ | Statins do not increase the rate of bleeding among warfarin users | Basic Clin Pharmacol Toxicol | Aug | 29504234 |
| Ogawa Y | Maternal exposure to benzodiazepine and risk of preterm birth and low birth weight: a case-control study using a claims database in Japan | Asia Pac Psychiatry | Sep | 29314716 |
| Martín-Merino E | Risk of venous thromboembolism among users of different anti-osteoporosis drugs: a population-based cohort analysis including over 200,000 participants from Spain and the UK | Osteoporos Int | Feb | 29199359 |
| Vekeman F | Retrospective cohort study comparing the risk of severe hepatotoxicity in hospitalized patients treated with echinocandins for invasive candidiasis in the presence of confounding by indication | BMC Infect Dis | 29-Aug | 30157797 |
| Dorman RM | The use of perioperative ketorolac in the surgical treatment of pediatric spontaneous pneumothorax | J Pediatr Surg | Mar | 28728827 |
| Bishwakarma R | Metformin use and health care utilization in patients with coexisting chronic obstructive pulmonary disease and diabetes mellitus | Int J Chron Obstruct Pulmon Dis | 20-Mar | 29551895 |
| Reinau D | Glucocorticoids and the risk of peptic ulcer bleeding: case-control analysis based on Swiss claims data | Drug Saf | Jul | 29468603 |
| Urpilainen E | Prognosis of ovarian cancer in women with type 2 diabetes using metformin and other forms of antidiabetic medication or statins: a retrospective cohort study | BMC Cancer | 28-Jul | 30055585 |
| Liao YT | Dosage of methylphenidate and traumatic brain injury in ADHD: a population-based study in Taiwan | Eur Child Adolesc Psychiatry | Mar | 28856464 |
| Fang SY | Excess mortality in children born to opioid-addicted parents: a national register study in Taiwan | Drug Alcohol Depend | 1-Feb | 29247973 |
| Meyers KJ | Evaluation of dystonia in children and adolescents treated with atomoxetine within the Truven MarketScan database: a retrospective cohort study | Expert Opin Drug Saf | May | 29625537 |
| Roetker NS | All-cause mortality risk with direct oral anticoagulants and warfarin in the primary treatment of venous thromboembolism | Thromb Haemost | Sep | 30103250 |
| Alzahrani T | Preoperative antibiotics and cardiovascular implantable electronic device infection: a cohort study in veterans | Pacing Clin Electrophysiol | Nov | 30221380 |
| Bettiol A | Pattern of use and long-term safety of tyrosine kinase inhibitors: a decade of real-world management of chronic myeloid leukemia | Clin Drug Investig | Sep | 30043130 |
| Xing S | Risk of diabetes hospitalization or diabetes drug intensification in patients with depression and diabetes using second-generation antipsychotics compared to other depression therapies | Prim Care Companion CNS Disord | 10-May | 29873957 |
| Verma AA | Fixed-dose combination antihypertensive medications, adherence, and clinical outcomes: a population-based retrospective cohort study | PLoS Med | Jun | 29889841 |
| Shah S | Comparative effectiveness of direct oral anticoagulants and warfarin in patients with cancer and atrial fibrillation | Blood Adv | 13-Feb | 29378726 |
| Ridolfi L | Increased frequency of acute reactions to iodinated contrast media in cancer patients treated with anti-ctla-4 immunomodulatory antibodies | Med Hypotheses | Oct | 30122486 |
| Amin A | A real-world observational study of hospitalization and health care costs among nonvalvular atrial fibrillation patients prescribed oral anticoagulants in the U.S. Medicare population | J Manag Care Spec Pharm | May | 32347184 |
| Hunnicutt JN | Commonly initiated opioids and risk of fracture hospitalizations in United States nursing homes | Drugs Aging | Oct | 30187291 |
| Lin SP | Risk of diabetes mellitus in HIV-infected patients receiving highly active antiretroviral therapy: a nationwide population-based study | Medicine (Baltimore) | Sep | 30200166 |
| Singh JA | Allopurinol and the risk of incident peripheral arterial disease in the elderly: a US Medicare claims data study | Rheumatology (Oxford) | 1-Mar | 29106674 |
| Coupland C | Antidepressant use and risk of adverse outcomes in people aged 20-64 years: cohort study using a primary care database | BMC Med | 8-Mar | 29514662 |
| Coleman CI | Effectiveness and safety of rivaroxaban versus warfarin in patients with unprovoked venous thromboembolism: a propensity-score weighted administrative claims cohort study | Thromb Res | Aug | 29883907 |
| Owosho AA | Medication-related osteonecrosis of the jaw: an update on the memorial sloan kettering cancer center experience and the role of premedication dental evaluation in prevention | Oral Surg Oral Med Oral Pathol Oral Radiol | May | 29580668 |
| Forslund T | Stroke and bleeding with non-vitamin k antagonist oral anticoagulant or warfarin treatment in patients with non-valvular atrial fibrillation: a population-based cohort study | Europace | 1-Mar | 28177459 |
| Leonard CE | Comparative safety of sulfonylureas and the risk of sudden cardiac arrest and ventricular arrhythmia | Diabetes Care | Apr | 29437823 |
| Lee HF | The effectiveness and safety of low-dose rivaroxaban in Asians with non-valvular atrial fibrillation | Int J Cardiol | 15-Jun | 29559181 |
| Kido T | The relationship between high-dose corticosteroid treatment and mortality in acute respiratory distress syndrome: a retrospective and observational study using a nationwide administrative database in Japan | BMC Pulm Med | 7-Feb | 29415701 |
| Effron MB | One-year clinical effectiveness comparison of prasugrel with ticagrelor: results from a retrospective observational study using an integrated claims database | Am J Cardiovasc Drugs | Apr | 29222628 |
| Huang CY | Renin-angiotensin-aldosterone blockade reduces atrial fibrillation in hypertrophic cardiomyopathy | Heart | Aug | 29371376 |
| Arnold FW | Macrolide therapy is associated with lower mortality in community-acquired bacteraemic pneumonia | Respir Med | Jul | 29957272 |
| Lin TT | Single and dual antiplatelet therapy in elderly patients of medically managed myocardial infarction | BMC Geriatr | 5-Apr | 29621983 |
| Yendamuri S | Risk and benefit of neoadjuvant therapy among patients undergoing resection for non-small-cell lung cancer | Eur J Cardiothorac Surg | 1-Mar | 29253122 |
| Gauci ML | Occurrence of type 1 and type 2 diabetes in patients treated with immunotherapy (anti-pd-1 and/or anti-ctla-4) for metastatic melanoma: a retrospective study | Cancer Immunology Immunotherapy | Aug | 29808365 |
| Tseng CH | Metformin and risk of developing nasopharyngeal cancer in patients with type 2 diabetes mellitus | Metabolism | Aug | 29729227 |
| Smith I | Do statins increase the risk of esophageal conditions? Findings from four propensity score-matched analyses | Clin Drug Investig | Feb | 29081029 |
| Grossman A | Treatment with levothyroxin in subclinical hypothyroidism is associated with increased mortality in the elderly | Eur J Intern Med | Apr | 29174213 |
| Hsu CW | Comparison of the effectiveness of brand-name and generic antipsychotic drugs for treating patients with schizophrenia in Taiwan | Schizophr Res | Mar | 28629889 |
| Steele SR | Evaluation of healthcare use and clinical outcomes of alvimopan in patients undergoing bowel resection: a propensity score-matched analysis | Dis Colon Rectum | Dec | 30312222 |
| Yimgang DP | Angiotensin converting enzyme inhibitors and interstage failure in infants with hypoplastic left heart syndrome | Congenit Heart Dis | Jul | 30019493 |
| Bedard NA | Does preoperative opioid use increase the risk of early revision total hip arthroplasty? | J Arthroplasty | Jul | 29452972 |
| Unni S | Comparative effectiveness of once-weekly glucagon-like peptide-1 receptor agonists with regard to 6-month glycaemic control and weight outcomes in patients with type 2 diabetes | Diabetes Obes Metab | Feb | 28862808 |
| Kang M | Effects of aspirin, nonsteroidal anti-inflammatory drugs, statin, and cox2 inhibitor on the developments of urological malignancies: a population-based study with 10-year follow-up data in Korea | Cancer Res Treat | Jul | 29081218 |
| Arana A | Variation in cardiovascular risk related to individual antimuscarinic drugs used to treat overactive bladder: a UK cohort study | Pharmacotherapy | Jun | 29723926 |
| Cheung KS | Long-term proton pump inhibitors and risk of gastric cancer development after treatment for helicobacter pylori: a population-based study | Gut | Jan | 29089382 |
| Bang UC | The relationship between the use of statins and mortality, severity, and pancreatic cancer in Danish patients with chronic pancreatitis | Eur J Gastroenterol Hepatol | Mar | 29309396 |
| Weir MR | Evaluation of clinical outcomes among nonvalvular atrial fibrillation patients treated with rivaroxaban or warfarin, stratified by renal function | Clin Nephrol | May | 29231162 |
| Hung YM | Hydroxychloroquine may be associated with reduced risk of coronary artery diseases in patients with rheumatoid arthritis: a nationwide population-based cohort study | Int J Clin Pract | May | 29691971 |
| Van de Ven LI | Association between use of antidepressants or benzodiazepines and the risk of subsequent fracture among those aged 65+ in the Netherlands | Osteoporos Int | Nov | 30112636 |
| Sonpavde G | Taxane chemotherapy vs antiandrogen agents as first-line therapy for metastatic castration-resistant prostate cancer | BJU Int | Jun | 29388324 |
| Zakiyah N | Antidepressant use during pregnancy and the risk of developing gestational hypertension: a retrospective cohort study | BMC Pregnancy Childbirth | 29-May | 29843629 |
| Etminan M | Risk of hair loss with different antidepressants: a comparative retrospective cohort study | Int Clin Psychopharmacol | Jan | 28763345 |
| Boursi B | Chronic therapy with selective serotonin reuptake inhibitors and survival in newly diagnosed cancer patients | Eur J Cancer Care (Engl) | Jan | 28252230 |
| Dave CV | Comparative risk of lipophilic and hydrophilic statins on incident depression: a retrospective cohort study | J Affect Disord | 1-Oct | 29936394 |
| Kuo YJ | Evaluation of nephroprotection of silymarin on contrast-induced nephropathy in liver cirrhosis patients: a population-based cohort study | Medicine (Baltimore) | Sep | 30212956 |
| Chan CW | Glitazones and alpha-glucosidase inhibitors as the second-line oral anti-diabetic agents added to metformin reduce cardiovascular risk in type 2 diabetes patients: a nationwide cohort observational study | Cardiovasc Diabetol | 24-Jan | 29368615 |
| Pan ML | Statin use and the risk of dementia in patients with stroke: a nationwide population-based cohort study | J Stroke Cerebrovasc Dis | Nov | 30087076 |
| Urpilainen E | The role of metformin and statins in the incidence of epithelial ovarian cancer in type 2 diabetes: a cohort and nested case-control study | BJOG | Jul | 29412502 |
| Hsu CY | Adherence to hydroxychloroquine improves long-term survival of patients with systemic lupus erythematosus | Rheumatology (Oxford) | 1-Oct | 29931367 |
| Adimadhyam S | Risk of amputations associated with SGLT2 inhibitors compared to DPP-4 inhibitors: a propensity-matched cohort study | Diabetes Obes Metab | Dec | 29971914 |
| Lee H | Assessing the risk of type 2 diabetes mellitus among children and adolescents with psychiatric disorders treated with atypical antipsychotics: a population-based nested case-control study | Eur Child Adolesc Psychiatry | Oct | 29460164 |
| Landi SN | No increase in risk of acute myocardial infarction in privately insured adults prescribed proton pump inhibitors vs histamine-2 receptor antagonists (2002-2014) | Gastroenterology | Mar | 29122546 |
| Batey M | Direct oral anticoagulants do not worsen traumatic brain injury after low-level falls in the elderly | Surgery | Oct | 30098813 |
| Lee YR | Association of antituberculosis treatment and lower risk of hyperlipidemia in Taiwanese patients: a population-based case-control study | In Vivo | Jan-Feb | 29275298 |
| Nagata N | Therapeutic endoscopy-related GI bleeding and thromboembolic events in patients using warfarin or direct oral anticoagulants: results from a large nationwide database analysis | Gut | Oct | 28874418 |
| Cho SK | Impact of anti-rheumatic treatment on cardiovascular risk in Asian patients with rheumatoid arthritis | Semin Arthritis Rheum | Feb | 28863826 |
| Yoshida S | Prenatal and early-life antibiotic use and risk of childhood asthma: a retrospective cohort study | Pediatr Allergy Immunol | Aug | 29604125 |
| Heo JH | Increased fracture risk with furosemide use in children with congenital heart disease | J Pediatr | Aug | 29753543 |
| Cheng YY | Effect of an increased dosage of statins on spinal degenerative joint disease: a retrospective cohort study | BMJ Open | 8-Feb | 29439066 |
| Lee YM | Inhaled corticosteroids in COPD and the risk of lung cancer | Int J Cancer | 1-Nov | 29943812 |
| Keller SF | Statin use and mortality in gout: a general population-based cohort study | Semin Arthritis Rheum | Dec | 29801703 |
| Kyvernitakis I | The tamoxifen paradox-influence of adjuvant tamoxifen on fracture risk in pre- and postmenopausal women with breast cancer | Osteoporos Int | Nov | 30032359 |
| Weycker D | Effectiveness and safety of apixaban versus warfarin as outpatient treatment of venous thromboembolism in U.S. Clinical practice | Thromb Haemost | Nov | 30357780 |
| Abe Y | Donepezil is associated with decreased in-hospital mortality as a result of pneumonia among older patients with dementia: a retrospective cohort study | Geriatr Gerontol Int | Feb | 29139192 |
| Liang CY | Flunarizine and the risk of parkinsonism in a newly diagnosed type 2 diabetic population in Taiwan: a nested case-control study | J Clin Neurosci | Apr | 29396059 |
| Amin A | Real-world comparison of all-cause hospitalizations, hospitalizations due to stroke and major bleeding, and costs for non-valvular atrial fibrillation patients prescribed oral anticoagulants in a US health plan | J Med Econ | Mar | 29047304 |
| Ortiz de Landaluce L | Gabapentin and pregabalin and risk of atrial fibrillation in the elderly: a population-based cohort study in an electronic prescription database | Drug Saf | Dec | 29956217 |
| Lin SM | Proton pump inhibitor use and the risk of osteoporosis and fracture in stroke patients: a population-based cohort study | Osteoporosis Int | Jan | 29032384 |
| Yang TY | Preoperative treatment with 5alpha-reductase inhibitors and the risk of hemorrhagic events in patients undergoing transurethral resection of the prostate - a population-based cohort study | Clinics (Sao Paulo) | 12-Mar | 29538495 |
| Calip GS | Tumor necrosis factor-alpha inhibitors and risk of non-Hodgkin lymphoma in a cohort of adults with rheumatologic conditions | Int J Cancer | 1-Sep | 29603214 |
| Arinze N | The effect of statin use and intensity on stroke and myocardial infarction after carotid endarterectomy | J Vasc Surg | Nov | 29685507 |
| Jung HH | Blood pressure-related risk among users versus nonusers of antihypertensives: a population-based cohort in Korea | Hypertension | Jun | 29686015 |
| Abrahami D | Incretin-based drugs and the incidence of colorectal cancer in patients with type 2 diabetes | Epidemiology | Mar | 29283894 |
| Oguro H | Retrospective analysis of argatroban in 353 patients with acute noncardioembolic stroke | J Stroke Cerebrovasc Dis | Aug | 29706441 |
| Teng CJ | Impact of anthracyclines on diabetes mellitus development in b-cell lymphoma patients: a nationwide population-based study | Clin Drug Investig | Jul | 29633159 |
| Anyanwagu U | Effect of adding GLP-1RA on mortality, cardiovascular events, and metabolic outcomes among insulin-treated patients with type 2 diabetes: a large retrospective UK cohort study | Am Heart J | Feb | 29421011 |
| Danielsson KC | Hypertensive pregnancy complications in women with epilepsy and antiepileptic drugs: a population-based cohort study of first pregnancies in Norway | BMJ Open | 24-Apr | 29691249 |
| Sandini M | Intraoperative dexamethasone decreases infectious complications after pancreaticoduodenectomy and is associated with long-term survival in pancreatic cancer | Ann Surg Oncol | Dec | 30298316 |
| Malik S | Sodium valproate and clozapine induced neutropenia: a case control study using register data | Schizophr Res | May | 28882687 |
| Kim SC | No difference in cardiovascular risk of tocilizumab versus abatacept for rheumatoid arthritis: a multi-database cohort study | Semin Arthritis Rheum | Dec | 29673963 |
| Nakaharai K | Early prophylactic antibiotics for severe acute pancreatitis: a population-based cohort study using a nationwide database in Japan | J Infect Chemother | Sep | 29909051 |
| Tzeng NS | Anti-herpetic medications and reduced risk of dementia in patients with herpes simplex virus infections-a nationwide, population-based cohort study in Taiwan | Neurotherapeutics | Apr | 29488144 |
| Real J | Safety of cilostazol in peripheral artery disease: a cohort from a primary healthcare electronic database | BMC Cardiovasc Disord | 8-May | 29739318 |
| Mii S | Cilostazol improves wound healing in patients undergoing open bypass for ischemic tissue loss: a propensity score matching analysis | Ann Vasc Surg | May | 29455014 |
| **Published in 2021** | |  |  |  |
| Abdel-Qadir H | The association between anticoagulation and adverse outcomes after a positive SARS-CoV-2 test among older outpatients: A population-based cohort study | Thromb Res | 13-Dec | 35149396 |
| Kobayashi K | The comparison of the kidney effects of dipeptidyl peptidase 4 inhibitors and glucagon-like peptide 1 agonist-administered concomitant with sodium-glucose cotransporter 2 inhibitors in Japanese patients with type 2 diabetes mellitus and chronic kidney disease | J Diabetes Res | 21-Dec | 35028319 |
| Hermann M | Androgen deprivation therapy and the risk for inguinal hernia: An observational nested case control study | Am J Mens Health | Nov-Dec | 34918553 |
| Ray AW | Association of rivaroxaban vs apixaban with major ischemic or hemorrhagic events in patients with atrial fibrillation | JAMA | 21-Dec | 34932078 |
| Herrera AV | Effects of prescription opioid use on traumatic brain injury risk in older adults | J Head Trauma Rehabil | Sep-Oct | 34489389 |
| Jones DA | The use of novel oral anticoagulants compared to vitamin K antagonists (warfarin) in patients with left ventricular thrombus after acute myocardial infarction | Eur Heart J Cardiovasc Pharmacother | 21-Sep | 32730627 |
| Sun Y | Effectiveness of the recombinant zoster vaccine in adults aged 50 and older in the United States: A claims-based cohort study | Clin Infect Dis | 15-Sep | 33580245 |
| Boikos C | Relative effectiveness of adjuvanted trivalent inactivated influenza vaccine versus egg-derived quadrivalent inactivated influenza vaccines and high-dose trivalent influenza vaccine in preventing influenza-related medical encounters in us adults ≥65 years during the 2017-2018 and 2018-2019 influenza seasons | Clin Infect Dis | 7-Sep | 33605977 |
| Tepper SJ | Effectiveness of erenumab and onabotulinumtoxinA on acute medication usage and health care resource utilization as migraine prevention in the United States | J Manag Care Spec Pharm | Sep | 33998825 |
| Chang CJ | Efficacy and Safety of Ticagrelor vs. Clopidogrel in East Asian Patients with Acute Myocardial Infarction: A Nationwide Cohort Study | Clin Pharmacol Ther | Feb | 32767756 |
| Kim DH | Frailty and clinical outcomes of direct oral anticoagulants versus warfarin in older adults with atrial fibrillation : a cohort study | Ann Intern Med | Sep | 34280330 |
| Herzig SJ | Relative risks of adverse events among older adults receiving opioids versus NSAIDs after hospital discharge: A nationwide cohort study | PLoS Med | 27-Sep | 34570810 |
| Davies F | Real-world comparative effectiveness of triplets containing bortezomib (B), carfilzomib (C), daratumumab (D), or ixazomib (I) in relapsed/refractory multiple myeloma (RRMM) in the US | Ann Hematol | Sep | 33970288 |
| Casale T | Real-world effectiveness of mepolizumab in patients with severe asthma and associated comorbidities | Ann Allergy Asthma Immunol | Sep | 34038773 |
| Xie J | Association of tramadol vs codeine prescription dispensation with mortality and other adverse clinical outcomes | JAMA | Oct | 34665205 |
| Rajamäki TJ | Is the preoperative use of antidepressants and benzodiazepines associated with opioid and other analgesic use after hip and knee arthroplasty? | Clin Orthop Relat Res | 1-Oct | 33982976 |
| Liao CT | The association between ivabradine and adverse cardiovascular events in acute decompensated HFrEF patients | ESC Heart Fail | Oct | 34327853 |
| Gueta I | Pregnancy outcomes following gestational exposure to papaverine: An observational comparative study | Br J Clin Pharmacol | Oct | 33675044 |
| Cao L | Neoadjuvant endocrine therapy as an alternative to neoadjuvant chemotherapy among hormone receptor-positive breast cancer patients: pathologic and surgical outcomes | Ann Surg Oncol | Oct | 34342757 |
| Butler AM | Risk of antibiotic treatment failure in premenopausal women with uncomplicated urinary tract infection | Pharmacoepidemiol Drug Saf | Oct | 33783918 |
