# Supplementary material for: Data source profile reporting by studies that use routinely collected health data to explore the effects of drug treatment
Source: BMC Med Res Methodol. 2023 Apr 20;23:95. doi: 10.1186/s12874-023-01922-8 (PMC10120171; doi:10.1186/s12874-023-01922-8)
Supplement: Supplementary file 2 — Supplementary Material 2 [file 12874_2023_1922_MOESM2_ESM.docx]

**Supplementary table 2 Reporting characteristics of data sources of 20 studies published in 2021**

| **Reporting item** | **Total (n = 20)** |
| --- | --- |
| **Linkage between data sources, n (%)** | 5(20.0) |
| Reporting the methods of linkage, n (%) | 3(60.0) |
| **Type of data source reported, n (%)** | 20(100.0) |
| **Name of database reported, n (%)** | 16(80) |
| **Whether the name of database include the type of data source, n (%)** | |
| Yes | 5(25.0) |
| Partly^*^ | 3(15.0) |
| Unclear^†^ | 0(0.0) |
| Not include | 12(60.0) |
| **Coverage of data source, n (%)** | 18(90.0) |
| **Categories of Specific country, n (%)** | 19(95.0) |
| **Data resource^‡^, n (%)** | 11(55.0) |
| **Data collected^§^, n (%)** | 8(40.0) |
| **Timeframe of database, n (%)** | 10(50.0) |
| **Population coverage, n (%)** | 11(55.0) |

^*^ Study involved multiple databases, but only a part of database contained information related to the type of data source

^†^ The name of the data source containing wording which did not clarify the type of data source, such as “clinical practice”, “health”, and “health information”

^‡^ Data resource included inpatients records, outpatients records, prescription records, etc.

^§^ Data collected such as demographics, diagnosis, laboratory and microbiology tests, prescription, operation, etc.
