# Supplementary figures and images for: Data source profile reporting by studies that use routinely collected health data to explore the effects of drug treatment
Source: BMC Med Res Methodol. 2023 Apr 20;23:95. doi: 10.1186/s12874-023-01922-8 (PMC10120171; doi:10.1186/s12874-023-01922-8)

**Supplementary figure 1. Flow diagram showing the process for identifying eligible studies
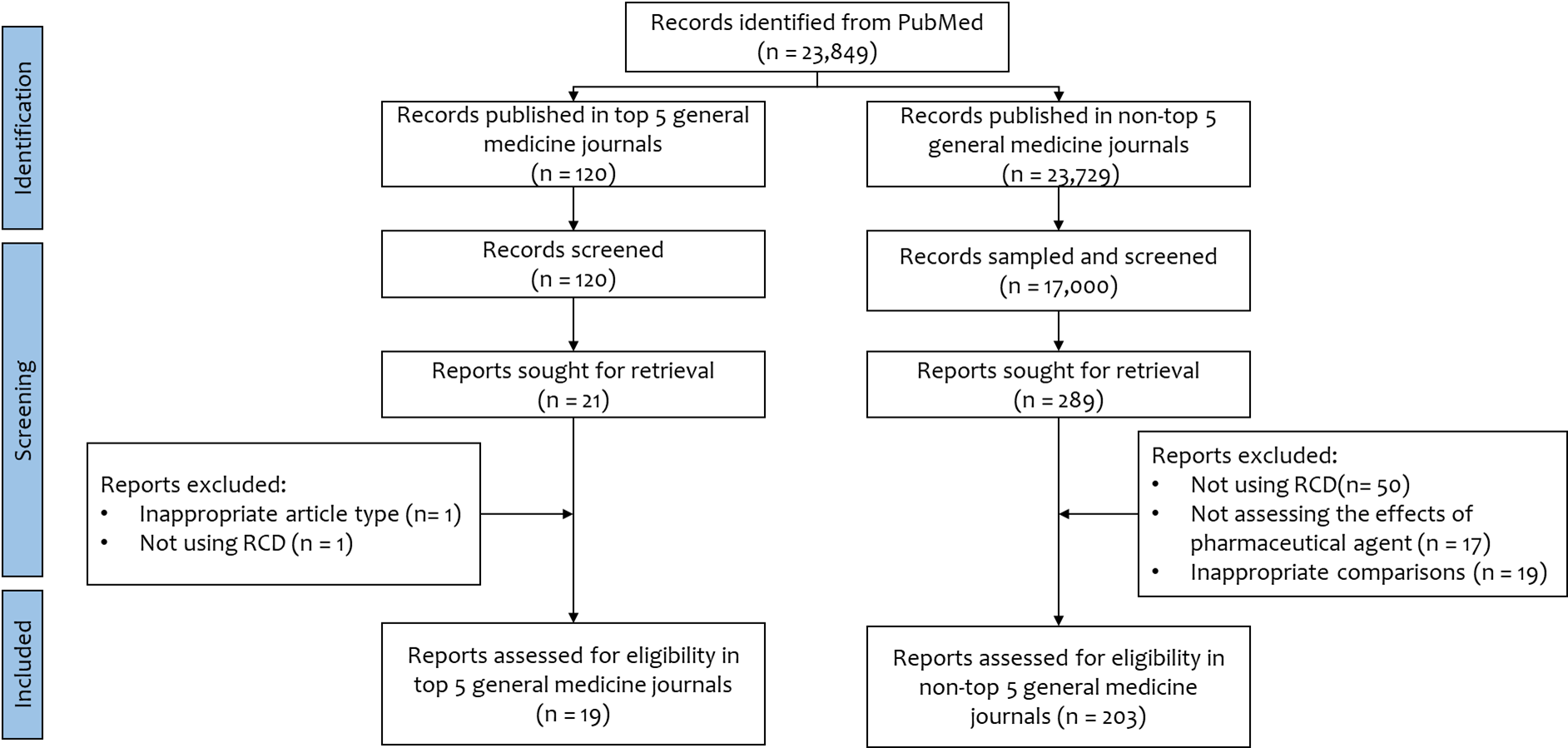
**

Supplement: Supplementary file 3 — Supplementary Material 3 [file 12874_2023_1922_MOESM3_ESM.docx]
